# Genetic Diversity of NHE1, Receptor for Subgroup J Avian Leukosis Virus, in Domestic Chicken and Wild Anseriform Species

**DOI:** 10.1371/journal.pone.0150589

**Published:** 2016-03-15

**Authors:** Markéta Reinišová, Jiří Plachý, Dana Kučerová, Filip Šenigl, Michal Vinkler, Jiří Hejnar

**Affiliations:** 1 Institute of Molecular Genetics, Academy of Sciences of the Czech Republic, Videnska 1083, CZ-14220, Prague 4, Czech Republic; 2 Charles University in Prague, Faculty of Science, Department of Zoology, Vinicna 7, CZ-12844, Prague 2, Czech Republic; National Institute of Allergy and Infectious Diseases, UNITED STATES

## Abstract

J subgroup avian leukosis virus (ALV-J) infects domestic chicken, jungle fowl, and turkey and enters the host cell through a receptor encoded by *tvj* locus and identified as Na+/H+ exchanger 1 (NHE1). The resistance to ALV-J in a great majority of examined galliform species was explained by deletions or substitutions of the critical tryptophan 38 in the first extracellular loop of NHE1, and genetic polymorphisms around this site predict the susceptibility or resistance of a given species or individual. In this study, we examined the NHE1 polymorphism in domestic chicken breeds and documented quantitative differences in their susceptibility to ALV-J *in vitro*. In a panel of chicken breeds assembled with the aim to cover the maximum variability encountered in domestic chickens, we found a completely uniform sequence of NHE1 extracellular loop 1 (ECL1) without any source of genetic variation for the selection of ALV-J-resistant poultry. In parallel, we studied the natural polymorphisms of NHE1 in wild ducks and geese because of recent reports on ALV-J positivity in feral Asian species. In anseriform species, we demonstrate a specific and highly conserved critical ECL1 sequence without any homologue of tryptophan 38 in accordance with the resistance of duck cells to prototype ALV-J. Last, we demonstrated that the new Asian strains of ALV-J have not evolved their envelope glycoprotein to the entry the duck cells. Our results contribute substantially to the current discussion of possible heterotransmission of ALV-J and its spill-over into the wild ducks and geese.

## Introduction

Avian leukosis virus subgroup J (ALV-J) was described as the prototype isolate HPRS-103 in commercial meat-type chickens in England [1]. The complete nucleotide sequence of HPRS-103 showed that ALV-J arose by recombination of an exogenous ALV with an endogenous *env* sequence [2,3]. HPRS-103 induces myelocytomatosis and a low incidence of other tumors after long latent periods but it can acquire the *myc* oncogene and develop into an acutely transforming retrovirus [4]. Comparison of various ALV-J isolates indicates rapid evolution so that antibodies to HPRS-103 do not neutralize later variants (see [5] for the review). Correspondingly, highly variable sequences were found in the *env* regions responsible for the host range and antigenic properties of US field strains [6]. Furthermore, new isolates induce a broader spectrum of neoplastic lesions, particularly erythroblastosis and cholangioma, in addition to myelocytomatosis [7]. This diversification of ALV-J strains historically coincided with its global spread, which started as sporadic outbreaks in Japan and resulted in a patchy but permanent occurrence of ALV-J in several Asian countries, particularly in China (see [8] for the review). Evolution of ALV-J can gain a new source of variability by recombination with B ALV subgroups [9,10] or endogenous retrovirus ALV-E [11]. These events may have triggered the spread of ALV-J from broilers to commercial layer chicken breeds.

Description of both vertical and early horizontal transmission of ALV-J in broiler breeder chickens [12,13] resulted in effective eradication schemes and successful elimination of ALV-J from breeding flocks in Europe and in the United States; on the other end, in China and other Asian countries, ALV-J is common predominantly in layer flocks and its eradication remains a major challenge (see [8] for the review). It is because of less rigorous control of poultry industry and widespread distribution of deeply divergent ALV-J strains with a broad range of pathogenesis.

ALV-J infects a very limited range of galliform species including domestic chicken, jungle fowls, and turkey [14]. The resistance to ALV-J in a great majority of galliform species has been explained by polymorphisms in the cell surface receptor for ALV-J, the Na^+^/H^+^ exchanger NHE1 [15], where deletions or substitutions of the critical tryptophan 38 correlate with resistance [16]. Quite recently, however, a few cases of ALV-J positivity were reported in species previously described as resistant. First, grey partridge (*Perdix perdix*) kept together with black-bone Silkie chickens contracted infection with a new strain of ALV-J and developed myelocytomas, lymphocytomas, erythroblastosis, and hemangiomas [17]. The host range extension was associated with mutations and deletions within hypervariable regions 1 and 2 [17]. Second, several wild passeriform and anseriform migratory species were identified as positive in a wide screen of samples collected in Northeastern China over the last three years [18,19]. Again, the ALV-J isolates from wild ducks were genetically distant from European and US strains whereas they belonged to the lineage of layer chicken ALV-J isolates. Although the incidence of ALV-J positivity was very low, these findings suggest that multiple ALV-J strains penetrate into the populations of wild birds or even circulate among them and there is a peril of establishing a natural reservoir of ALV-J.

The host range of ALV-J is very limited because of receptor incompatibility described in many galliform species for the HPRS103 strain [16]. In gaining a new foothold, the virus might be more successful in species with receptors more similar to those of susceptible species. *Vice versa*, the historical spread from meat-type to layer chicken flocks indicates some differences in the susceptibility of various chicken breeds to ALV-J. Such quantitative differences in sensitivity to A and B subgroups of ASLV were previously attributed to the genetic make-up of respective inbred chicken lines and multiple polymorphisms in *tva* and *tvb* loci were identified [20,21]. Therefore, the knowledge of ALV-J receptor variants in resistant species and receptor polymorphisms in chickens is urgently needed to better understand the past evolution and spread of ALV-J as well as to predict its capacity for infecting populations of wild birds. In this study, we examined the susceptibility to ALV-J in a panel of inbred chicken lines and analyzed the critical part of NHE1, the receptor for ALV-J, in a variety of chicken breeds and in multiple species of wild ducks and geese.

## Materials and Methods

### Animals, origin of DNA samples and cell cultivation

Chicken embryo fibroblasts (CEF) for the ALV-J infection experiment were obtained from a panel of eight inbred chicken lines maintained at the Institute of Molecular Genetics, Prague [22]. The panel includes lines BLi, CB, H6, L15, M, P, S, and WA. CEFs were prepared from 10-day-old embryos and grown in a mixture of two parts of Dulbecco’s modified Eagle’s medium and one part of F-12 medium supplemented with 8% fetal calf serum, 2% chicken serum, and 1 x antibiotic-antimycotic solution (Sigma) in a 5% CO_2_ atmosphere at 40°C. In parallel to the infection experiment, CEFs were used for isolation of total RNA.

In the same way, the total RNA of domestic duck (*Anas platyrhynchos domestica*) was isolated from duck embryo fibroblasts (DEF) of inbred Khaki Campbell line maintained at the Institute of Molecular Genetics [23,24]. DEFs were prepared from 12-day-old embryos and grown in the same medium as CEFs.

Embryo fibroblasts from all other species (turkey, Japanese quail, gray partridge, common pheasant, guinea fowl, and chukar) were prepared and cultured as decribed previously [16]. Total RNA of gray partridge (*Perdix perdix*) was isolated from cultured embryo fibroblasts.

The samples of outbred chicken breeds included either DNA or RNA of Araucana, Barnevelder Bantam, Booted Bantam, Brahma, Chabo (Japanese Bantam), Czech Gold Brindled Hen, Frizzle, La Flèche, Minorca, Naked Neck, Orloff, Padovana Bantam, Phoenix, Polish Bantam, Rosecomb Bantam, Sebright Bantam, Shamo, Silkie, and Yokohama kept by hobby breeders. The DNA was prepared from blood or muscles and RNAs from a mix of embryonic immunological tissues. The samples for RNA isolation have been stored in in RNA Later at -80°C and RNeasy Mini Kit (Qiagen) was used for RNA isolation. The samples of blood or muscles have been stored -20°C until isolation of DNA using the DNeasy Blood & Tissue Kit (Qiagen) and the resulting DNA was stored in ethanol at -20°C later on.

The DNAs of wild anseriform species were obtained from feather pulp samples. The panel of wild ducks and geese included twenty species kept in ZOO Hluboká, Czech Republic: northern pintail (*Anas acuta*), Eurasian teal (*Anas crecca*), yellow-billed teal (*Anas flavirostris*), Baikal teal (*A*. *formosa*), Eurasian wigeon (*Anas penelope*), garganey (*Anas querquedula*), gadwall (*Anas strepera*), graylag goose (*Anser anser*), emperor goose (*Anser canagicus*), lesser white-fronted goose (*Anser erythropus*), common pochard (*Aythia ferina*), tufted duck (*Aythia fuligula*), ferruginous duck (*Aythia nyroca*), red-breasted goose (*Branta ruficollis*), common goldeneye (*Bucephala clangula*), tundra swan (*Cygnus columbianus*), rosy-billed pochard (*Netta peposaca*), red-crested pochard (*Netta rufina*), ruddy shelduck (*Tadorna feruginea*), and common shelduck (*Tadorna tadorna*). Wing feathers containing pulp were collected, the shafts with pulp were cutt off and stored on dry ice for DNA isolation. RNA isolation from feather pulp samples was inefficient in our hands.

### Subgroup J reporter vector construction

GFP-transducing replication-competent vectors of J subgroup were constructed on the basis of RCASBP(A)GFP as described previously [16]. RCASBP(A)GFP was digested by KpnI and StuI (both New England Biolabs), and the 1,853-bp internal fragment containing the 3´ end of *pol* and the whole *env* was replaced by corresponding KpnI-StuI fragments from ALV-J strains HPRS103 ([2]; GenBank Z46390), ADOL7501 (GenBank AY027920), PDRC-59831 ([25]; GenBank KP284572), and ZB110604-5 ([17]; GenBank KC841156). In addition, we included the ALV-J *env* sequence WB11016j isolated from Eurasian wigeon in China ([19]; GenBank JX570795). HPRS103 env sequence was obtained as KpnI-StuI fragment from the molecular clone kindly provided by V. Nair, Institute for Animal Health, Compton, United Kingdom. ADOL7501, PDRC-59831, ZB110604-5, and WB11016j *env* sequences were synthesized (Integrated DNA Technologies) and cloned using the conserved KpnI site and introduced BsaBI site, sequence GATAGGAATC, 3´ to the termination codon. The resulting constructs were denoted RCASBP(J)GFP, RCASBP(J_ADOL_)GFP, RCASBP(J_PDRC_)GFP, RCASBP(J_ZB_)GFP, and RCASBP(J_WB_)GFP, respectively.

### Virus propagation and infection of CEFs

Susceptibility to ALV-J was assessed using the J subgroup recombinant vectors described above. Infectious virus was produced in DF-1 cells [26] transfected with plasmid DNA. Virus stocks were harvested on day 9 or 10 posttransfection (p.t.). The cell supernatants were cleared of debris by centrifugation at 2,000 x g for 10 min at 10°C, and aliquoted viral stocks were stored at -80°C. The virus titer was determined by terminal dilution and subsequent infection of DF-1 cells and reached 10^6^ infection units (IU) per ml. CEFs of inbred lines were seeded at a density of 2.5 x 10^4^ per well in a 24-well plate. 8 h after seeding, the cells were infected with decreasing amount of virus 2.5 x 10^5^ IU (multiplicity of infection, MOI = 10), 2.5 x 10^4^ (MOI = 1), 2.5 x 10^3^ (MOI = 0.1), 2.5 x 10^2^ (MOI = 0.01) or 2.5 x 10^1^ (MOI = 0.001) of RCAS(J)GFP. The virus was applied in 0.25 ml medium for 1 h. The percentage of GFP-positive cells was quantitated by fluorescence-activated cell sorting (FACS) using an LSRII analyzer (Becton, Dickinson) on days 1, 2, 3,4, and 7 postinfection (p.i.). The cells were trypsinized, washed in culture medium, and resuspended in Hoechst stain solution (Sigma) before the analysis.

### DNA/RNA preparation and amplification of NHE1 ECL1

Feather shafts were stored frozen on dry ice. When possible, the feather pulp was squeezed from the shaft and incubated and rolled in 1 ml of 1% SDS, 0.25 M EDTA pH 8.0, and 1 mg/ml proteinase K at 55°C over night. Then, after addition of 300 μg RNase A, incubation was continued at 37°C for 1 hour. The insoluble rests of the shafts were discarded and high molecular DNAs were purified from the lysate using conventional phenol:chloroform extraction and ethanol precipitation. Total RNA from cultivated CEFs, DEFs, and partridge fibroblasts was prepared using the TRI Reagent (Sigma).

We amplified the NHE1 gene either from RNA or DNA samples. RNA from CEFs, DEFs, gray partridge fibroblasts, Araukana, La Flèche, Orloff, Padovana Bantam, Phoenix, Polish Bantam and Yokohama was reverse transcribed from 1 μg total RNA with Moloney murine leukemia virus reverse transcriptase and oligo(dT)15 primers (both from Promega). We amplified the N-terminal part of the NHE1 coding sequence, which contains all predicted transmembrane domains and extra- and intracellular loops, using the forward primer chTVJ1L (5´-CTTCCCTGGGCTCTGCTG-3´, nucleotides 25 to 42 downstream of the ATG initiation codon) and reverse primer chTVJ6R (5´-CAGGAACTGCGTGTGGATCTC-3´, complementary to nucleotides 1,537 to 1,557 of the chNHE1). The following PCR conditions were used: 98°C for 180 s, 35 cycles of 98°C for 10 s, 67.6°C for 30 s, and 72°C for 100 s, and terminal extension at 72°C for 7 min with Taq polymerase (TaKaRa). The resulting PCR product of 1,533 bp in length was treated with ExoSAP-IT (USB) and then directly sequenced from the chTVJ5´RACE1 primer (5´-TCATCAGGCAGGCCAGCAGGAT-3´, complementary to nucleotides 301 to 322) using a BigDye Terminator v3.1 cycle sequencing kit (PE Applied Biosystems).

chNHE1 from DNA samples of Barnevelder Bantam, Booted Bantam, Brahma, Chabo (Japanese Bantam), Czech Gold Brindled Hen, Frizzle, Minorca, Naked Neck, Rosecomb Bantam, Sebright Bantam, Shamo, and Silkie was amplified using primers chTVJ1L and chTVJARV (5´-TGTGGTGCTGTTTGTTCAGC-3´ complementary to nucleotides 183 to 202 of the chNHE1 cDNA). The PCR conditions used were: 98°C for 180 s, 35 cycles of 98°C for 10 s, 67°C for 30 s, and 70°C for 30 s, and terminal extension at 72°C for 7 min with Taq polymerase (TaKaRa). The resulting fragment of 178 bp containing the variable part of ECL1 was sequenced from the chTVJARV primer.

For amplification of anseriform NHE1, we designed primers duckTVJ1L (5´- CGTGGGTGCTGCTGGTGTTG-3´) and duckARV (5´-GGTTGGCCGCCTGCTTGTTC-3´), which are the duck analogs of chTVJ1L and chTVJARV. The PCR conditions were the same as for chicken DNA samples and the resulting fragment was sequenced from the duckARV primer.

### Ethics Statement

All animal samples were obtained according to protocols approved by the Committee for Animal Welfare and Protection at the Institute of Molecular Genetics. Samples of outbred chickens were obtained from fancy breeders in the Czech Republic based on the informed consent. Collection of muscle microsamples from living birds was performed by skilled staff according to protocols approved by the Veterinary Institute of the Czech Republic. Although invasive, this procedure is not very painful, does not require any analgesis, and does not represent any harm for the birds. ZOO Hluboka approved collection of feathers from birds. In order to minimize the stress associated with manipulation the captive but still shy birds, the collection was done at the same time with routine vaccination against botulism and all birds were manipulated by skilled animal keepers.

## Results

### Inbred lines of chicken differ in their susceptibility to ALV-J

In a previous study [16], we described the *in vitro* susceptibility to ALV-J in a panel of eight galliform species. Three breeds of domestic chicken were included as a positive control and we observed a slower course of ALV-J infection in the cells of H6 inbred line in comparison with other chicken breeds and sensitive species, jungle fowl and turkey. This difference was most striking in an experiment with low multiplicity of infection (MOI, [16]). In order to describe the full range of sensitivity to ALV-J and to find chicken breeds with potential polymorphisms in the ECL1 of the *tvj* locus, we infected the panel of eight inbred chicken lines with a RCAS(J)GFP vector and followed the percentage of GFP+ cells for seven days p.i. The results of infection with MOI = 1 and MOI = 10 are shown in Table 1. We observed efficient infection with ca. 95% of GFP+ cells with MOI = 10 in L15, BLi, and M inbred lines. In the rest of inbred lines, the susceptibility was significantly lower and the less susceptible H6 line reached only 65.4% of GFP+ cells with MOI = 10. Infection at MOI = 1 resulted in lower percentages of GFP+ cells with most susceptible L15 line reaching 71% of GFP+ cells and less susceptible H6 line stopping at 51.8% of GFP+ cells (Table 1). The order of the rest of inbred lines was not necessarily the same in both experiments with different MOIs.

**Table 1:** The time course of RCAS(J)GFP infection at MOI = 1/MOI = 10 in embryo fibroblasts of inbred chicken lines.

% of GFP-positive cells in days p.i.^a^^,^^b^					
Inbred line	1	2	3	4	7
L15	32.2/39.0	63.0/71.6	63.4/81.6	71.3/85.4	71.0/95.7^c^
BLi	22.9/43.4	58.4/76.8	63.2/78.6	69.6/83.3	56.0/96.5^c^
M	18.2/40.4	50.4/71.8	56.6/77.5	62.6/79.3	55.8/94.0^c^^,^^e^
WA	32.1/42.3	47.8/67.4	61.7/66.4	63.4/75.7	60.1/77.1^e^
P	21.7/41.7	50.1/66.4	56.9/65.7	61.2/73.2	63.8/82.4
CB	27.9/39.4	58.2/65.8	63.8/68.4	65.8/74.3	67.7/86.0
S	34.4/40.6	63.9/66.4	67.1/67.6	69.8/70.0	63.5/73.6^d^
H6	22.6/28.9	49.4/54.7	53.3/61.1	59.6/63.3	51.8/65.4^d^

^a^The natural autofluorescence of mock-infected cells was 0.05% of GFP+ cells.

^b^The data show the averages of three parallels, SDE was calculated for each group of three and reached only low values between ± 0.36 and ± 2.38 (not shown). The data of day 7 p.i., MOI = 10 were statistically analyzed using a paired Student´s test.

^c,d^Values denoted c differ significantly (P < 0.01) from values denoted d.

^e^Values denoted e differ significantly, P < 0.05.

**Fig 1:**
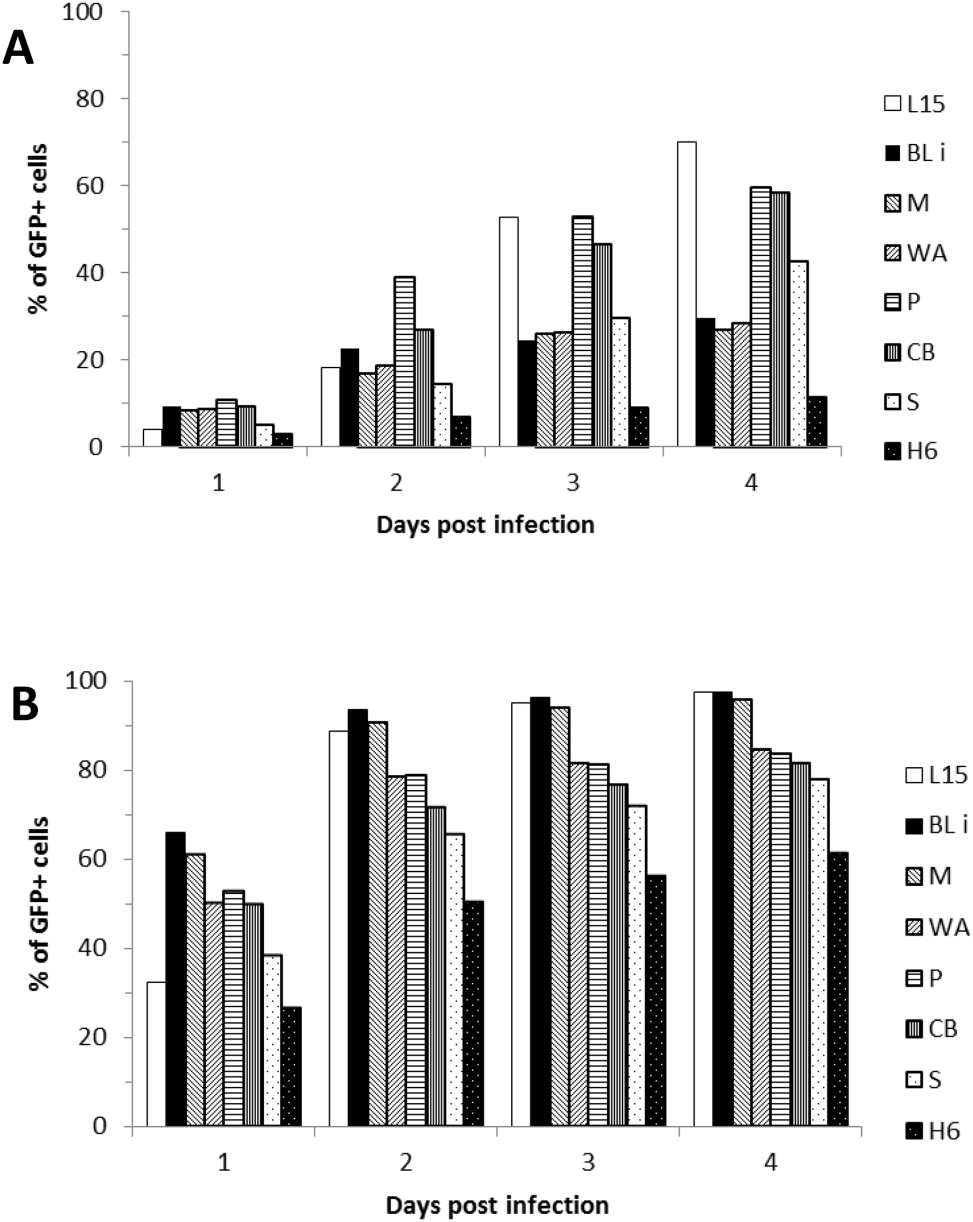
Differences in the susceptibility to ALV-J among embryo fibroblasts from inbred chicken lines. Infections were done with decreasing MOIs of RCAS(J)GFP and GFP+ cells were examined by FACS four d. p.i. MOIs of 0.1 (A) and 10 (B) are shown. The data represent a typical experiment done in one parallel for each inbred line and each time.

In order to further demonstrate the differences in susceptibility of chicken inbred lines to ALV-J, we performed an experiment with very low MOIs. We infected CEFs of L15, CB, M, WA, H6, and Bli lines with gradually diluted RCAS(J)GFP so that the MOI decreased from 10 to 0.001 and the percentage of GFP+ cells was measured four days p.i. (Fig 1). Whereas MOIs 10 and 1 resulted in mild differences among the inbred lines, MOI = 0.1 discriminated significantly the more sensitive L15 and CB lines on the one hand and the less sensitive M, WA, Bli, and H6 lines on the other hand. These differences were even more striking with MOIs 0.01 and 0.001, which were close to the terminal dilution of the virus. In summary, we observed differences in the susceptibility to ALV-J among the inbred lines of chicken with different genetic backgrounds.

### Lack of polymorphism in the first extracellular loop of NHE1 in domestic chicken

The demonstrated differences in susceptibility to ALV-J might be caused by amino-acid substitutions in the cellular receptor chNHE1, as was already shown for subgroup B and A receptors, Tvb and Tva, respectively [20,21]. In NHE1, the first extracellular loop in the N-terminal part concentrates almost all the interspecific variability, and a single amino-acid substitution or deletion of W38 discriminates between susceptibility and resistance to ALV-J [16]. We therefore sequenced the region of ECL1 within the chNHE1 gene of all eight chicken inbred lines and looked for any changes in their amino-acid sequence. In all eight chicken inbred lines, the ECL1 amino-acid sequence was uniform corresponding to the wild-type described previously in domestic chicken and red jungle fowl [15,16]. We found only a few synonymous nucleotide polymorphisms within ECL1 (data not shown). Next, we checked the amino-acid sequence of ECL1 in a panel of 20 chicken breeds including both traditional Western breeds and the geographically restricted and fancy breeds from Europe and Asia. One of the breeds is of the ancestral black skin phenotype and we assume that this panel covers a substantial part of the genetic variability present in domestic chicken. Again, we found no ECL1 polymorphism in these chicken breeds. This corresponds with the fact that none of the domestic chicken breeds was referred to be resistant to ALV-J infection. We conclude that chNHE1 is highly conserved with no amino-acid polymorphism within the ECL1 region.

### Screening for polymorphisms in ECL1 of NHE1 in wild duck species

**Fig 2:**
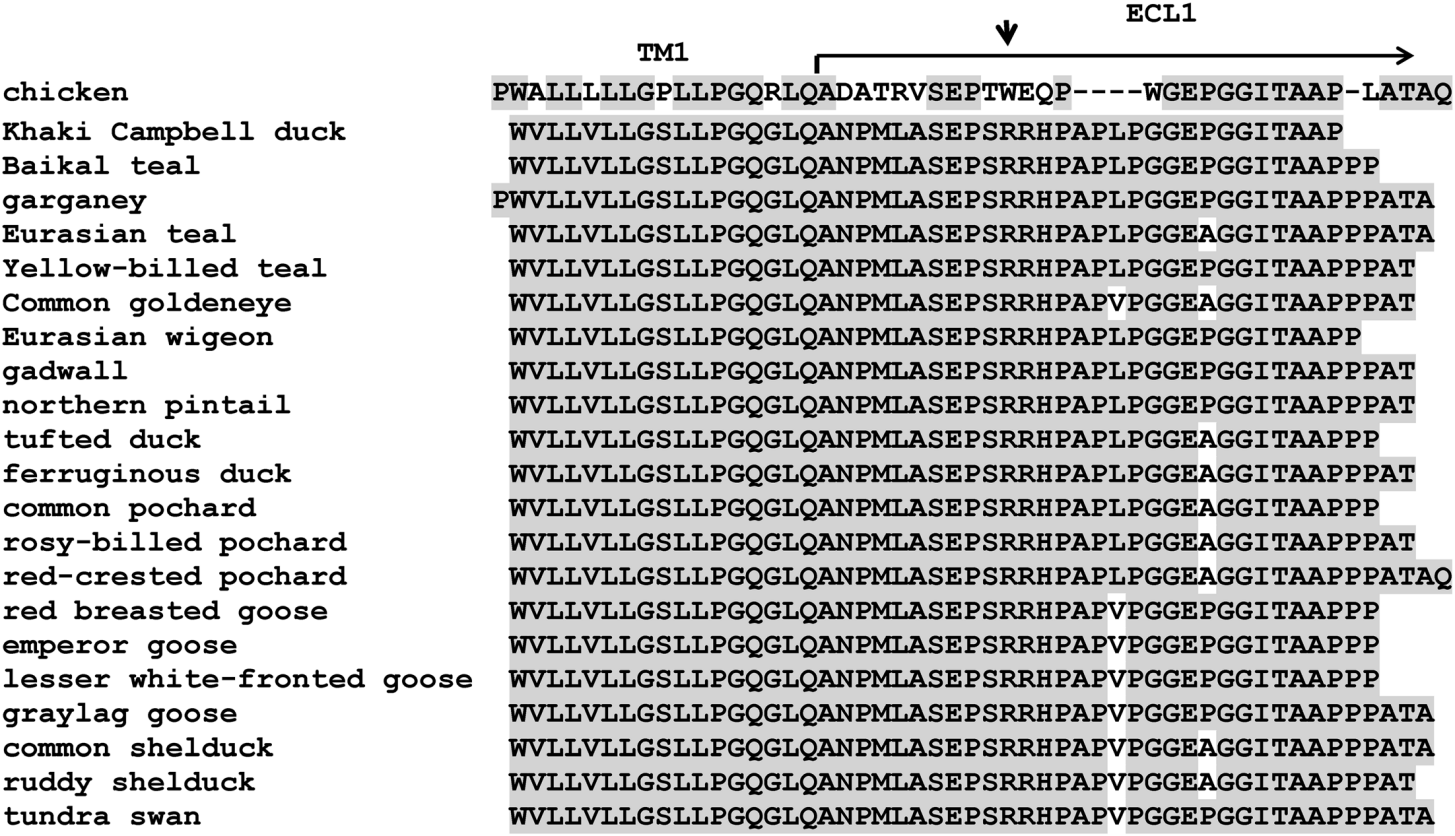
Alignment of the left part of ECL1 and adjacent TM1 of NHE1 in the duck and goose species. The deduced amino-acid sequences of ECL1 and corresponding chNHE1 amino-acids 11 to 58 are compared and aligned. The border between ECL1 and putative transmembrane domain TM1 is depicted by horizontal arrow. The W38 amino-acid residue in chNHE1 is shown as a vertical arrow. Amino acids matching the consensus sequence of anseriforms are on a gray background.

Next, we amplified and sequenced ECL1 in twenty wild species of anseriforms, mostly Eurasian, with particular interest in the species previously documented as ALV-J positive in China [18,19]. As expected, the N-terminal part of ECL1 is highly divergent between chicken and duck, with just a few amino-acid residues conserved (Fig 2). No tryptophan that could play the role of critical W38 in chNHE1 is present in duck ECL1. On the other hand, there is a good correspondence within the first transmembrane domain and the putative extent of ECL1 matches that of chNHE1. Starting from G43 of chNHE1, the ECL1 region is well conserved, with few mismatches between the chicken and duck. Although ECL1 is highly polymorphic in galliform species [16], we found just two polymorphic amino-acids among the inspected anseriform species (Fig 2). First, there is either proline or alanine at the position corresponding to P45 in chNHE1. Proline occurs in genus *Anas*, with the exception of Eurasian teal, and in genera *Anser*, *Branta* and *Cygnus*. Alanine appears in genera *Aythia*, *Bucephala*, *Netta*, and *Tadorna* and in Eurasian teal. Second, the 17th amino-acid of anseriform ECL1 is either leucine in genera *Anas*, *Aythia*, and *Netta*, or valine in genera *Anser*, *Branta*, *Bucephala*, *Cygnus*, and *Tadorna* (Fig 2). In addition, we observed a minimum of amino-acid polymorphisms in heterozygous state, which suggests very limited intraspecific NHE1 variability in ducks. We found also several synonymous nucleotide substitutions within the sequenced part of ECL1. The full sequences with all polymorphisms are given in S1 (geese) and S2 (ducks) Figs. In cocnlusion, anseriform NHE1 is uniform within the ECL1 region and lacks any tryptophan residue that could mimick the W38 critical for susceptibility to ALV-J in chicks.

### Deletion of the critical W38 in the gray partridge NHE1

**Fig 3:**
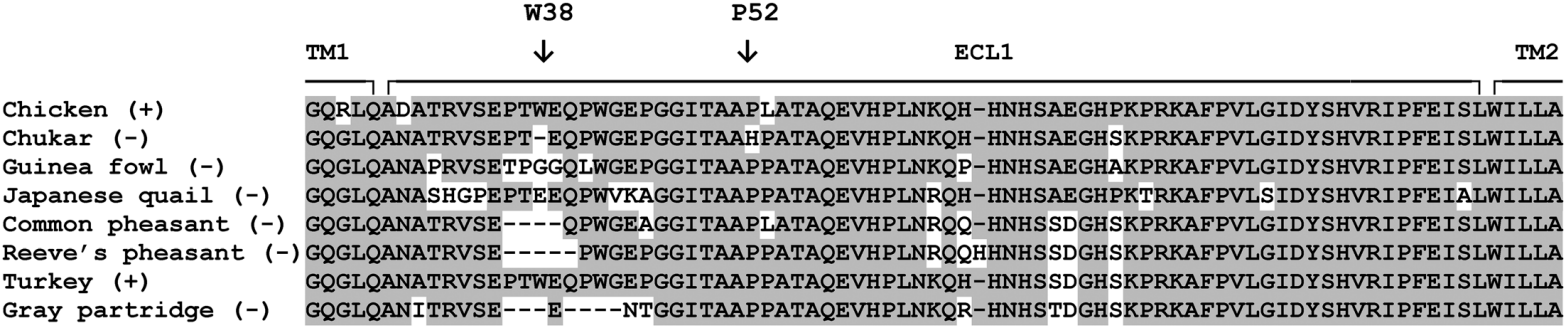
NHE1 allele in gray partridge contains deletion of W38 resembling the ALV-J resistant species in galliforms. The deduced gray partridge amino-acid sequence of ECL1 corresponding to chNHE1 amino-acids 23 to 104 are aligned to the sequences of eight galliform species analyzed previously (Kučerová et al., 2013). The susceptibility or resistance of galliform species is denoted (+) or (-), respectively. The borders between ECL1 and putative transmembrane domains TM1 and TM2 are shown. The W38 and P52 amino acid residues are denoted by vertical arrows. Amino acids matching the consensus sequence are on a gray background.

The recent report of gray partridge infection with ALV-J [17] is at odds with previous testing the gray partridge as resistant to the HPRS103 strain of ALV-J [16]. We therefore analyzed the cDNA sequence and deduced the amino-acid sequence of the gray partridge NHE1 in the ECL1 region. As shown in the ECL1 alignment of several galliform species in Fig 3, we found deletion of amino-acids 36 to 38 including the critical W38 residue. In addition, we observed deletion of amino-acids 40 to 43 and several substitutions, A30I, EP44-45NT, H66R, AE71-72TD, and P75S, in comparison to the chNHE1. The partial cDNA sequence of NHE1 of gray partridge and corresponding amino-acid sequence is given in S3 Fig. Some of these amino-acid changes are shared with other galliform species. In summary, gray partridge NHE1 exemplifies a typical allele resistant to HPRS103.

### Resistance of duck and gray partridge cells to J subgroup ALV vectors

In order to test directly the possible host extension of variant ALV-J strains, we constructed J subgroup RCAS vectors transducing the GFP reporter and infected the duck and chicken cells in culture. In addition to the already examined *env* gene of the prototypic ALV-J strain HPRS130, we constructed RCAS vectors with the *env* gene of the American strain ADOL7501, the last American isolate PDRC-59831 [25], Chinese isolate from gray partridge ZB110604-5 [17], and the J-subgroup *env* sequence amplified from Eurasien wigeon sampled in northeast China [19]. All *env* sequences provided the functioning envelope to the vectors that produced infectious and replication-competent virus particles. The RCASBP(J_PDRC_)GFP vector produced the highest titer of virus particles. All J-subgroup RCAS vectors efficiently infected chicken and turkey cells in culture (Table 2). The RCASBP(J_PDRC_)GFP infected 97% of chicken DF-1 cells within 4 days after infection, the remaining J-subgroup RCAS vectors were less efficient (Table 2) resembling the HPRS103 *env* at similar multiplicity (Table 1). Turkey embryo fibroblasts were less susceptible to all J-subgroup RCAS vectors with 92.6% of infected cells after infection with RCASBP(J_PDRC_)GFP vector. We observed only marginal infection of duck embryo fibroblasts with RCASBP(J_ADOL_)GFP and RCASBP(J_WB_)GFP (Table 2). In both cases, the percentages of infected cells were only slightly above the bacground of mock-infected cells and by three orders of magnitude lower than that of chicken cells. Gray partridge embryo fibroblasts were completely resistant to all J-subgroup vectors (Table 2). These results show that the new variants of ALV-J, even those isolated from wild ducks and gray partridge, maintain the original species specificity and their *env* genes did not adapt towards new hosts.

**Table 2:** Host range of new J subgroup RCAS vectors in avian species^a^.

Species/Virus	RCASBP(J_PDRC_)GFP	RCASBP(J_ADOL_)GFP	RCASBP(J_WB_)GFP	RCASBP(J_ZB_)GFP
Chicken (DF-1)	91.1 (97.1)	46.7 (61.7)	69.4 (80.5)	47.6 (79.3)
Turkey	75.4 (92.6)	38.3 (50.4)	43.2 (60.1)	26.6 (54.0)
Domestic duck	<0.05^b^	<0.05 (0.08)	<0.05 (0.07)	<0.05
Japanese quail (QT6)	<0.05	<0.05	<0.05	<0.05 (0.08)
Gray partridge	<0.05	<0.05	<0.05	<0.05
Common pheasant	<0.05	<0.05	<0.05	<0.05
Guinea fowl	<0.05	<0.05	<0.05	<0.05
Chukar	<0.05	<0.05	<0.05 (0.11)	<0.05 (0.07)

^a^The embryo fibroblasts of a given species, or DF-1 and QT6 cell lines, were infected with multiplicity of infection = 1. The percentage of GFP-positive cells two or four (in parentheses) days post infection are given as an average of two parallels.

^b^A value of 0.05% of GFP-positive cells represents the autofluorescence of mock-infected cells.

## Discussion

Our results demonstrate that all examined chicken lines with different genetic background can be infected with ALV-J and display only slight differences in their susceptibility to ALV-J. The time course of infection by RCAS vectors has proven to be a good correlate of susceptibility to various ASLV subgroups [16,20,21]. Our original RCASBP(J)GFP vector was constructed on the basis of the prototype ALV-J strain, HPRS-103. Because of the great differences between the classic and new Asian ALV-J isolates, however, we are aware that our findings are limited to HPRS103. Here, we have constructed new J subgroup RCAS vectors with *env* genes from American and Chinese ALV-J isolates and all of them efficiently infect chicken cells in culture. Further studies should compare the susceptibility of inbred chicken lines to these variant ALV-J isolates. It should be noted that our model reflects only the receptor-virus interaction and neglects other parts of the virus restriction.

In our panel of inbred chicken lines and two close-bred strains, the differences in susceptibility to ALV-J are more pronounced at the low multiplicity of infection (MOI). Interestingly, the susceptibility to ALV-J, expressed by the proportion of GFP positive cells in particular chicken line, does not fully correlate at the low and high MOI (Table 1). In sharp contrast to these observed differences in the susceptibility to RCAS-J infection, we did not detect any polymorphisms in the critical sequence of Tvj receptor, the chNHE1. In a panel of outbred chicken breeds, we found the identical sequence of the Tvj receptor corroborating the fact that no resistant chicken line has been described until now. The panel of outbred chicken breeds was assembled with the aim to cover the maximum of variability encountered in domestic chicken. It is, however, difficult to infer the genetic kinship and origin of chicken breeds from modern genetic data alone [27] and we admit that some polymorphisms within the ECL1 region of chNHE1 as a source of the breeding for ALV-J resistance might be found in the future. The absence of chNHE1 polymorphisms suggests that the slight differences in susceptibility to ALV-J observed between inbred chicken lines could be attributed to other mechanisms of retrovirus restriction.

NHE1 is a house-keeping gene and it is conceivable that its sequence is highly conserved due to purifying selection. On the other hand, we have found previously that *tvj* alleles from galliform species contain remarkable polymorphisms within the ECL1 of the chNHE1 molecule. Especially, the critical W38 distinguishes ALV-J susceptible and resistant species [16]. The importance of tryptophan (or other aromatic amino-acid) residues followed by an acidic amino-acid residue was described also for Tva, Tvb, ecotropic MLV receptor, and HIV receptor [28,29]. The absence of W38 in several wild galliform species suggests that amino-acid substitutions within ECL1 do not abrogate the function of NHE1 as Na+/H+ exchanger and W38 genome editing could be employed for preparation of ALV-J-resistant poultry.

In the last twenty years, we witnessed a rapid evolution of the ALV-J virus leading to strains pathogenic for the layer chickens. There are also signs of further increase in the host range. Grey partridge has been infected and repeated the ALV-J-induced pathology [17] and new strains of ALV-J were isolated from wild *passeriforms* and ducks [18,19]. Wild ducks and geese are of particular interest because they come into close contact with domestic poultry in China and migrate between their breeding and wintering areas. Therefore, they coud become a natural reservoir of ALV-J and spread it over long distances. In order to predict the species, that are at least in part susceptible to ALV-J, we screened a representative panel of *anseriform* species for the polymorphisms of the ECL1 part of the NHE1 molecule. In contrast to the situation in previously studied *galliform* species, we found quite a different N-terminal part of ECL1 with a minimum polymorphism among anseriform species (Fig 2). W38 is not present in any *anseriform* species. Because of the minimum interspecific polymorphism, we cannot expect meaningful intraspecific polymorphisms with exception of the position corresponding to P45 in chNHE1. Here, proline or alanine alternate in different species and this polymrphism occurs also among individuals of the same species. The described polymorphisms in amino-acids are also to be tested for the susceptibility to ALV-J. Even partial susceptibilty could explain the presence of ALV-J in some anseriform species. We can also hypothesize that the new ALV-J strains might have evolved toward an extended host range. Their glycoproteins might at least weekly recognise different part(s) of NHE1 or have adapted to a new cell surface receptor. This would be a continuation of the existing diversity in the ALV complex, whose closely related A—E subgroups adapted to various receptors related to low-density lipoprotein receptors, tumor necrosis factor receptors, and butyrophilins [30,31,32]. Our results, however, do not support speculations about the escape development of envelope glycoproteins in the new strains of ALV-J. Cultured duck cells tested almost refractory to the infection with RCAS vectors equiped with *env* genes from several American and Chinese ALV-J strains, which represent the ALV-J variability within both meat and layer chicken breeds. Only marginal virus infection was observed after long time (4 days) with envelopes from ADOL7501 and WB11016j. Having tested the *env* genes directly from the reported duck isolate WB11016j, we can conclude that the new ALV-J strains have not evolved their envelope glycoprotein to the entry the duck cells. Our results arise concerns if the reported findings of ALV-J in wild ducks [18,19] could be attributed to the low-efficiency infection observed. We should keep in mind that our analysis focused solely in env-receptor interaction and we did not tested the cells of respective wild duck species. Interference experiments with the prototype HPRS103 strain and the new Asian strains should be performed, and cells of the wild duck species should be tested for their susceptibility to ALV-J directly in culture.

In parallel to anseriform species, we analyzed the ECL1 of NHE1 in grey partridge previously shown positive for ALV-J [17]. We found deletion of several amino-acids including W38 (see alignment in Fig 3). This is in agreement wit previous finding that gray partridge is resistant to HPRS103 [16]. Again, we tried to infect gray partridge cells with J subgroup RCAS vectors with envelopes from various strains, but we did not observe any infection in this case even with *env* from the ZB110604-5 isolate. On the other hand, we observed marginal infection in japanese quail and in chukar with envelopes from the new Chinese ALV-J isolates. Our results contribute substantially to the current discussion of possible heterotransmission of ALV-J and potential spill-over into the wild populations of ducks and geese. In the future, it will be necessary to analyze virus propagation in duck cells and test the infection in cells of wild duck species.

## Supporting Information

S1 Fig The nucleotide alignment of the partial NHE1 sequence of eight geese species. Nucleotides matching the database sequence of domestic goose are on a gray background. The non-conserved nucleotides changing the amino-acid translation are in red. The first predicted amino-acid of the ECL1 is in green.
(DOCX)

S2 Fig The nucleotide alignment of the partial NHE1 sequence of 15 duck species. Nucleotides matching the database sequence of domestic duck are on a gray background. The non-conserved nucleotides changing the amino-acid translation are in red. The first predicted amino-acid of the ECL1 is in green. The color bacground denotes nucleotides heterozygous in examined individual of a given species. Red, T and C means W and R in Khaki Campbell duck and common goldeneye; yellow, G and C means W and C in Northern pintail, tufted duck, common pochard, and red-crested pochard; pink, G and C in rosy-billed pochard; turquoise, C and T in Eurasian wigeon and gadwall; violet, C and T means P and S in common goldeneye; green, G and T in yellow-billed teal; blue, C and G means A and P in yellow-billed teal and gadwall.
(DOCX)

S3 Fig Partial cDNA sequence of NHE1 of gray partridge and corresponding amino-acid sequence. (DOCX)
