# Supplementary material for: Genetic Diversity of NHE1, Receptor for Subgroup J Avian Leukosis Virus, in Domestic Chicken and Wild Anseriform Species
Source: PLoS One. 2016 Mar 15;11(3):e0150589. doi: 10.1371/journal.pone.0150589 (PMC4792377; doi:10.1371/journal.pone.0150589)
Supplement: S1 Fig — Nucleotides matching the database sequence of domestic goose are on a gray background. The non-conserved nucleotides changing the amino-acid translation are in red. The first predicted amino-acid of the ECL1 is in green. (DOCX) [file pone.0150589.s001.docx]

**W V  L L V  L L G S L L P G Q G L Q A N P M L A S E**

**Domestic goose TGGGTGCTGCTGGTGTTGTTGGGCTCGCTGCTCCCCGGGCAGGGCTTGCAGGCCAACCCCATGTTGGCTTCCGAG**

**Red breasted goose TGGGTGCTGCTGGTGTTGTTGGGCTCGCTGCTACCCGGGCAGGGCTTGCAGGCCAACCCCATGTTGGCTTCCGAG**

**Emperor goose TGGGTGCTGCTGGTGTTGTTGGGCTCGCTGCTCCCCGGGCAGGGCTTGCAGGCCAACCCCATGTTGGCTTCCGAG**

**Lesser w.f. goose TGGGTGCTGCTGGTGTTGTTGGGCTCGCTGCTCCCCGGGCAGGGCTTGCAGGCCAACCCCATGTTGGCTTCCGAG**

**Graylag goose TGGGTGCTgCTGGTGTTGTTGGGCTCGCTGCTCCCCGGGCAGGGCTTGCAGGCCAACCCCATGTTGGCTTCCGAG**

**Common shelduck TGGGTGCTGCTGGTGTTGCTGGGCTCGCTGCTACCCGGGCAGGGCTTGCAGGCCAACCCCATGTTGGCTTCCGAG**

**Ruddy shelduck TGGGTGCTGCTGGTGTTGCTGGGCTCGCTGCTACCCGGGCAGGGCTTGCAGGCCAACCCCATGTTGGCTTCCGAG**

**Tundra swan TGGGTGCTGCTGGTGTTGTTGGGCTCGCTGCTACCCGGGCAGGGCTTGCAGGCCAACCCTATGTTGGCTTCCGAG**

**P S R R H P A P V P G G E P G G I T A A P P P A T A**

**Domestic goose CCTTCCCGGAGACACCCGGCACCGGTACCGGGAGGGGAACCCGGGGGTATCACGGCCGCGCCGCCGCCGGCCACGGCG**

**Red breasted goose CCTTCCCGGAGACACCCGGCACCGGTACCGGGAGGGGAACCCGGGGGTATCACGGCCGCGCCGCCGCCG**

**Emperor goose CCTTCCCGGAGACACCCGGCACCGGTACCGGGAGGGGAACCCGGGGGTATCACGGCCGCGCCGCCGCCGGCCACG**

**Lesser w.f. goose CCTTCCCGGAGACACCCGGCACCGGTACCGGGAGGGGAACCCGGGGGTATCACGGCCGCGCCGCCGCCG**

**Graylag goose CCTTCCCGGAGACACCCGGCACCGGTACCGGGAGGGGAACCCGGGGGTATCACGGCCGCGCCGCCGCCG**

**Common shelduck CCTTCCCGGAGACACCCGGCACCGGTACCAGGGGGGGAAGCCGGGGGTATCACGGCCGCGCCGCCGCCGGCCACGGCG**

**Ruddy shelduck CCTTCCCGGAGACACCCGGCACCGGTACCGGGGGGGGAAGCCGGGGGTATCACGGCCGCGCCGCCGCCGGCCACGG**

**Tundra swan CCTTCCCGGAGACACCCGGCACCGGTACCGGGAGGGGAACCCGGGGGTATCACGGCCGCGCCGCCGCCGGCCACGGCG**

**Supplementary Figure 1**
