# Supplementary material for: Genetic Diversity of NHE1, Receptor for Subgroup J Avian Leukosis Virus, in Domestic Chicken and Wild Anseriform Species
Source: PLoS One. 2016 Mar 15;11(3):e0150589. doi: 10.1371/journal.pone.0150589 (PMC4792377; doi:10.1371/journal.pone.0150589)
Supplement: S2 Fig — Nucleotides matching the database sequence of domestic duck are on a gray background. The non-conserved nucleotides changing the amino-acid translation are in red. The first predicted amino-acid of the ECL1 is in green. The color bacground denotes nucleotides heterozygous in examined individual of a given species. Red, T and C means W and R in Khaki Campbell duck and common goldeneye; yellow, G and C means W and C in Northern pintail, tufted duck, common pochard, and red-crested pochard; pink, G and C in rosy-billed pochard; turquoise, C and T in Eurasian wigeon and gadwall; violet, C and T means P and S in common goldeneye; green, G and T in yellow-billed teal; blue, C and G means A and P in yellow-billed teal and gadwall. (DOCX) [file pone.0150589.s002.docx]

**W V  L L V L L G S L L P G Q G L Q A N P M L A S E**

**Domestic duck CGTGGGTGCTGCTGGTGTTGCTGGGCTCGCTGCTCCCCGGGCAGGGCTTGCAGGCCAACCCCATGTTGGCTTCCGAG**

**Khaki-Campbell duck CGTGGGTGCTGCTGGTGTTGTTGGGCTCGCTGCTCCCCGGGCAGGGCTTGCAGGCCAACCCCATGTTGGCTTCCGAG**

**Baikal teal CGTGGGTGCTGCTGGTGTTGCTGGGCTCGCTGCTCCCCGGGCAGGGCTTGCAGGCCAACCCCATGTTGGCTTCCGAG**

**Garganey CGTGGGTGCTGCTGGTGTTGCTGGGCTCGCTGCTCCCCGGCCAGGGCTTGCAGGCCAACCCCATGTTGGCTTCCGAG**

**Eurasian teal CGTGGGTGCTGCTGGTGTTGCTGGGCTCGCTGCTCCCCGGGCAGGGCTTGCAGGCCAACCCCATGTTGGCTTCCGAG**

**Yellow-billed teal CGTGGGTGCTGCTGGTGTTGCTGGGCTCGCTGCTCCCCGGGCAGGGCTTGCAGGCCAACCCCATGTTGGCTTCCGAG**

**Common goldeneye CGTGGGTGCTGCTGGTGTTGCTGGGCTCGCTGCTACCCGGGCAGGGCTTGCAGGCCAACCCCATGTTGGCTTCCGAG**

**Eurasian wigeon CGTGGGTGCTGCTGGTGTTGCTGGGCTCGCTGCTCCCCGGGCAGGGCTTGCAGGCCAACCCCATGTTGGCTTCCGAG**

**Gadwall CGTGGGTGCTGCTGGTGTTGTTGGGCTCGCTGCTCCCCGGGCAGGGCTTGCAGGCCAACCCCATGTTGGCTTCCGAG**

**Northern pintail CGTGGGTGCTGCTGGTGTTGCTGGGCTCGCTGCTCCCCGGGCAGGGCTTGCAGGCCAACCCCATGTTGGCTTCCGAG**

**Tufted duck CGTGGGTGCTGCTGGTGTTGCTGGGCTCGCTGCTACCCGGGCAGGGCTTGCAGGCCAACCCCATGTTGGCTTCCGAG**

**Ferruginous duck CGTGGGTGCTGCTGGTGTTGCTGGGCTCGCTGCTACCCGGGCAGGGCTTGCAGGCCAACCCCATGTTGGCTTCCGAG**

**Common pochard CGTGGGTGCTGCTGGTGTTGCTGGGCTCGCTGCTACCCGGGCAGGGCTTGCAGGCCAACCCCATGTTGGCTTCCGAG**

**Rosy-billed pochard CGTGGGTGCTGCTGGTGTTGCTGGGCTCGCTGCTACCCGGGCAGGGCTTGCAGGCCAACCCCATGTTGGCTTCCGAG**

**Red-crested pochard CGTGGGTGCTGCTGGTGTTGCTGGGCTCGCTGCTACCCGGGCAGGGCTTGCAGGCCAACCCCATGTTGGCTTCCGAG**

**P S R R H P A P L P G G E A G G I T A A P P P A T A**

**Domestic duck CCTTCCCGGAGACACCCGGCACCGCTACCGGGGGGGGAAGCCGGGGGTATCACGGCCGCGCCGCCGCCGGCCACGGC**

**Khaki-Campbell duck CCTTCCCGGAGACACCCGGCACCGCTACCGGGGGGGGAACCCGGGGGTATCACGGCCGCGCCGCCGCCGGCCACGGC**

**Baikal teal CCTTCCCGGAGACACCCGGCACCGCTACCGGGTGGGGAACCCGGGGGTaTCACGGCCGCGCCGCCGCCG**

**Garganey CCTTCCCGGAGACACCCGGCACCGCTACCGGGTGGGGAACCCGGGGGTATCACGGCCGCGCCGCCGCCGGCCACGGC**

**Eurasian teal CCTTCCCGGAGACACCCGGCACCGTTACCGGGGGGGGAAGCCGGGGGTATCACGGCCGCGCCGCCGCCGGCCACGGC**

**Yellow-billed teal CCTTCCCGGAGACACCCGGCACCGCTACCGGGGGGGGAACCCGGGGGTATCACGGCCGCGCCGCCGCCGGCCACGGC**

**Common goldeneye CCTTCCCGGAGACACCCGGCACCGGTACCGGGGGGGGAAGCCGGGGGTATCACGGCCGCGCCGCCGCCGGCCACG**

**Eurasian wigeon CCTTCCCGGAGACACCCGGCACCGCTACCGGGTGGGGAACCCGGGGGTATCACGGCCGCGCCACCGCC**

**Gadwall CCTTCCCGGAGACACCCGGCACCGCTACCGGGTGGGGAACCCGGGGGTATCACGGCCGCGCCACCGCCGGCCACGGC**

**Northern pintail CCTTCCCGGAGACACCCGGCACCGCTACCGGGTGGGGAACCCGGGGGTATCACGGCCGCGCCGCCGCCGGCCACGGC**

**Tufted duck CCTTCCCGGAGACACCCGGCACCGCTACCGGGTGGGGAAGCCGGGGGTaTCACGGCCGCGCCGCCGCCG**

**Ferruginous duck CCTTCCCGGAGACACCCGGCACCGCTACCGGGTGGGGAAGCCGGGGGTATCACGGCCGCGCCGCCGCCGGCCACGG**

**Common pochard CCTTCCCGGAGACACCCGGCACCGCTACCGGGTGGGGAAGCCGGGGGTaTCACGGCCGCGCCGCCGCCG**

**Rosy-billed pochard CCTTCCCGGAGACACCCGGCACCGCTACCGGGTGGGGAAGCCGGGGGTATCACGGCCGCGCCGCCGCCGGCCACGG**

**Red-crested pochard CCTTCCCGGAGACACCCGGCACCGCTACCGGGTGGGGAAGCCGGGGGTATCACGGCCGCGCCGCCGCCGGCCACGGC**

**Supplementary Figure 2**
