# Supplementary material for: Genetic Diversity of NHE1, Receptor for Subgroup J Avian Leukosis Virus, in Domestic Chicken and Wild Anseriform Species
Source: PLoS One. 2016 Mar 15;11(3):e0150589. doi: 10.1371/journal.pone.0150589 (PMC4792377; doi:10.1371/journal.pone.0150589)
Supplement: S3 Fig — (DOCX) [file pone.0150589.s003.docx]

**GCTCTGCTGCTGCTGCTGGGCCCGCTGCTTCCCGGCCAAGGTTTACAGGCCAACATCACTCGTGTTTCCGAAGAAAACACGGGGGGCATCACCGCCGCCCCCCCCGCCACGGCTCAGGAGGTTCACCCGCTGAACAAACAGCGGCACAACCACTCGACCGATGGCCATTCCAAACCCCGCAAAGCTTTCCCCGTGCTGGGCATCGATTATTCTCACGTCCGCATCCCCTTCGAGATCTCGCTCTGGATCCTGCTGGCCTGCTTGATGAAGATGGGCTTCCACGTGATGTCCTCAGTGTCCAAAGTGGTTCCCGAGAGCTGCCTGCTCATCGTGGTGGGCCTTCTGGTCGGCGGGCTCATTAAAGCAGTGGGCGAAAAGCCCCCCATCCTCAAATCAGACATCTTCTTCCTCTTCCTTCTCCCTCCCATCATTTTGGACGCCGGCTATTTCCTTCCCTTGCGCCAGTTCACGGAGAACCTGGGCACCATCCTCATCTTCGCCGTGGTGGGGACGCTCTGGAATGCTTTTTTCCTGGGGGGGCTGATGTATGCCGTGTGCCAGATCGGCGGCAGCGGCCTCAACCACATCGGCCTGCTGGCCAACCTGCTCTTCGGCAGCATCATCTCGGCCGTGGACCCGGTGGCAGTGCTGGCCGTCTTTGAGGAGATCCACATCAACGAGTTGCTGCACATCCTGGTCTTCGGGGAGTCCCTGCTGAACGACGCCGTCACGGTGGTCCTCTACCACCTTTTTGAGGAGTTTGCCTACTTTGAGCAAGTGACCATTATCGATATCATCCTTGGCTTCCTCAGCTTCTTCGCGGTGTCTCTGGGCGGCGTCTTCGTGGGCGTCATTTATGGGCTGATTGCTGCCTTCACGTCCCGCTTCACCTCCAACATCCGTGTCATCGAGCCCCTCTTCGTCTTCCTCTACAGCTACATGGCCTACCTCTCTGCTGAGCTCTTCCACCTCTCCGGCATCATGGCGCTCATCGCCTCCGGTGTGGTCATGCGGCCCTACGTGGAAGCCAACATCTCCCACAAGTCCCACACCACCATCAAGTATTTCCTCAAGATGTGGAGCAGCGTGAGCGAGACCCTCATCTTCATCTTCCTGGGTGTCTCCACCGTGGCTGGCCAGCACTACTGGAACTGGACCTTCGTCATCAGCACGCTGCTCTTCTGCCTCATCGCGAGGGTTTTAGGCGTCCTGGTCCTCACCTGGTTCATCAACAAATTCCGGATCGTGAAGCTGACACCA**

**AAGGATCAGTTCATCATTGCCTACGGGGGCCTGCGGGGAGCCATCGCCTTCTCCCTCTGCTACCTCCTCGACTACGAGCACTTTAACATGAGGGACATGTTCCTCACGGCCATCATCACCGTCATCTTCTTCACTGTCTTCGTGCAGGGCATGACCATCC**

**ALLLLLGPLLPGQGLQANITRVSEENTGGITAAPPATAQEVHPLNKQRHNHSTDGHSKPRKAFPVLGIDYSHVRIPFEISLWILLACLMKMGFHVMSSVSKVVPESCLLIVVGLLVGGLIKAVGEKPPILKSDIFFLFLLPPIILDAGYFLPLRQFTENLGTILIFAVVGTLWNAFFLGGLMYAVCQIGGSGLNHIGLLANLLFGSIISAVDPVAVLAVFEEIHINELLHILVFGESLLNDAVTVVLYHLFEEFAYFEQVTIIDIILGFLSFFAVSLGGVFVGVIYGLIAAFTSRFTSNIRVIEPLFVFLYSYMAYLSAELFHLSGIMALIASGVVMRPYVEANISHKSHTTIKYFLKMWSSVSETLIFIFLGVSTVAGQHYWNWTFVISTLLFCLIARVLGVLVLTWFINKFRIVKLTPKDQFIIAYGGLRGAIAFSLCYLLDYEHFNMRDMFLTAIITVIFFTVFVQGMTI**

**Supplementary Figure 3**
